# Complete genome sequence of *Cronobacter sakazakii* ES191 generated by PacBio sequencing

**DOI:** 10.1128/mra.00473-24

**Published:** 2024-11-08

**Authors:** Shuyan Wu, Ruy Jauregui, Paul Maclean, Gale Brightwell

**Affiliations:** 1 AgResearch Ltd., Grasslands Research Centre, Palmerston North, New Zealand; 2 Animal Health Laboratory, Biosecurity New Zealand, Ministry for Primary Industries, Wellington, New Zealand; 3 New Zealand Food Safety Science and Research Centre, Massey University, Palmerston North, New Zealand; Queens College Department of Biology, Queens, New York, USA

**Keywords:** PacBio, genomes, food-borne pathogens

## Abstract

We present the complete genome of *Cronobacter sakazakii* ES191, isolated from New Zealand in 2011 and known for its strong biofilm ability. The genome consists of a 4,309,604-base pair (bp) circular chromosome (56.9% GC) and a 165,613-bp circular plasmid (55.4% GC).

## ANNOUNCEMENT

Biofilms are of concern in both the food industry and medical field, as they can lead to contamination of food products and transmission of disease (1). Super biofilm-producing strains, in particular, present a challenge as they create resilient biofilms that are difficult to eradicate (2). By comprehending the genome, we can gain insights into the mechanisms driving strong biofilm formation, facilitating the development of strategies to mitigate biofilm-related risks and enhance food safety and public health. The genome of *Cronobacter sakazakii* ES191, known for its strong biofilm ability, is revealed in this investigation.

This bacterium, isolated from a dairy environment in 2011, was obtained and identified as *C. sakazakii* from the International Organization for Standardization -accredited commercial or research laboratories in New Zealand (3). A colony was purified by restreaking three times on tryptic soy agar (Fort Richard, NZ) and cultured in 10 mL of tryptic soy broth at 37°C overnight (18 h). High-molecular-weight genomic DNA (1.4 µg) was freshly extracted from 1 mL of overnight culture using a modified phenol–chloroform procedure (4). The genomic DNA was sheared using Covaris g-TUBE (Novogene, China) at 2,029 × *g* for 2 min to target ~10-kb fragments.

The ES191 genome was sequenced using the Pacific Biosciences Sequel II platform (Novogene, China). The SMRTbell genome library was prepared as instructed by the SMRTbell prep kit 2.0 procedure and checklist (Novogene, China). The ES191 genome was barcoded using the SMRTbell barcoded adapter plate 3.0 (Novogene, China), pooled with two other bacterial genomes, and sequenced in one SMRT (single-molecule real-time) cell, which was carried out with 120 pM on-plate concentration, Sequel II binding kit 2.0, diffusion loading type, chemistry 11.0. The PacBio Sequel II generated 30,726 polymerase reads with N50 = 98,549, which were partitioned to 163,315 subreads with N50 = 14,729. The software SMRTlink v11.0 was used to filter and process original sequencing results, with minLength 0, minReadScore 0.8 as parameters, for error correction and adapter trimming.

The sequence reads were assembled using Flye (version 2.9-b1768) with the “--pacbio-raw” parameter set (5). The assembled genome of ES191 isolate yielded two circular contigs. Contig 1 comprised a single chromosome [4,309,604 base pairs (bp), 56.9% G+C content], and Contig 2 comprised a single plasmid (165,613 bp, 55.4% G+C content). The ES191 isolate genome comprises a total length of 4,475,217 bp, spanning two fragments with an N50 fragment size of 4,309,604 bp. The mean coverage of the genome is 372. The genome annotation was performed by the NCBI Prokaryotic Genome Annotation Pipeline v6.1, resulting in the identification of 4,088 coding sequences, 85 tRNAs, and 22 rRNAs. The whole genome-based taxonomic analysis was performed using the Type (Strain) Genome Server (https://tygs.dsmz.de). The analysis confirmed the ES191 isolate as *Cronobacter sakazakii*, as validated by both 16S rRNA and genomic data.

## Data Availability

The strain ES191 genome sequence as output by the assembler has been deposited in the GenBank under accession numbers CP150985 and CP150984, BioProject number PRJNA948061, and BioSample number SAMN33874645, and the sequencing reads have been deposited in the SRA as accession SRR28576677.
